# The model of rat lipid metabolism disorder induced by chronic stress accompanying high-fat-diet

**DOI:** 10.1186/1476-511X-10-153

**Published:** 2011-08-28

**Authors:** Lin Manting, Zhou Haihong, Li Jing, Chen Shaodong, Liu Yihua

**Affiliations:** 1 Medical College of Xiamen University, No.168, Daxue Road, Xiamen Fujian Province-361005, China

**Keywords:** Lipid Metabolism Disorder, animal model, chronic stress, high-fat-diet

## Abstract

**Abstract Objective:**

To develop an animal model of Lipid Metabolism Disorder, which conforms to human clinical characteristic. Methods: There were 24 male Wistar rats that were randomly divided into 3 groups with 8 rats in each. They were group A (normal diet), group B (high-fat-diet), group C (chronic stress+ high-fat-diet). Group A was fed with normal diet, while group B and C were fed with high-fat-diet, going on for 55 days. From the 35th day, group B and C received one time of daily chronic stress, going on for 21 days. After that, the activities of the serum alanine aminotransferase (ALT) and aspartate aminotransferase (AST), and the levels of the serum triglyceride (TG), Cholesterol (Ch), high-density lipoprotein-Cholesterol (HDL-C) and liver TG were evaluated. Results: Compared with group A, the activities of the serum ALT and AST, and the levels of the serum CH, TG, HDL-C and liver TG were found to be markedly increased, when the level of HDL-C was markedly decreased in group B and C, and the results of group C was more obviously. Conclusion: Chronic stress and high-fat-diet have the synergistic action in rat's Lipid Metabolism Disorder. They lead to a model of Lipid Metabolism Disorder, which conforms to human clinical characteristic much better.

## 1. Introduction

Lipid Metabolism Disorder means the quality and the amount of the lipids and its metabolites in the blood and other tissue gets abnormal congenitally or acquired. It is one of the major risk factors of coronary heart disease, arteriosclerosis, fatty liver, diabetes B, hypertension and some other diseases[1,2]. Hence, reinforcing the prevention and treatment of Lipid Metabolism Disorder has critical clinical meaning. In the research of Lipid Metabolism Disorder, the building of the model is of great importance. The documents concerning the building of animal model in Lipid Metabolism Disorder research is quite few, so far. What is more, most of these documents only use only one pathogenic factor, such as high-fat-diet and the inducement of chemical substances in the model building [3,4]. However, this is different from the clinical characteristics of Lipid Metabolism Disorder. Therefore, establishing an animal model, which simulates the nosogenesis of human Lipid Metabolism Disorder, is important for the maintenance of this disease. Our research chooses the most common pathogenic factor of the modern life style -- chronic stress and high fat diet. This research use noise, light, temperature and some other stimulation to form chronic stress and use high-fat-diet, and then observe the impact on the lipid metabolism. So we can build the animal model, which has the characteristics of Lipid Metabolism Disorder.

## 2. Materials and methods

### 2.1 Materials and Animal

24 male Wistar rats, weight 193 ± 20 g, clean grade, bought from Shanghai Silaike Experimental Animal Co., Ltd, Animal License Key: SCXK (Hu) 2007_0005. Raised at Animal Experiment Center of Medical College of Xiamen University, have free access to water. 24 male Wistar rats were randomly divided into 3 groups with 8 rats in each. They were grouping A (normal diet), group B (high-fat-diet) and group C (chronic stress+ high-fat-diet). Group A was fed with normal diet, when group B and C was fed with high-fat-diet, going on for 55 days. The experiment was conducted in accordance with current Chinese legislation and standards of the Chinese College of Animal Experimentation. The use of animals in this study was approved by the ethics committee at Medical College of Xiamen University (protocol 4315 of 07/29/10).

### 2.2 High-fat-diet

High-fat-diet include 83.25% basal feed, 10% lard, 1.5% Clesterol, 0.2% NaTDC, 5% Sugar and 0.05% Propylthiouracil.

### 2.3 Chronic Stress Plan

Improved Katz Chronic Stress [5], design Chronic Stress Plan (Table 1), From the 36th day, group C received one time of daily chronic stress, going on for 21 days.

**Table 1:** Chronic Stress Plan Table

D36 Fasting	D37 Fasting	D38 Water Deprivation	D39 Night Lighting	D40 Day-Night Inversion	D41 Wet Feeding	D42 Water Deprivation
D43 Day-Night Inversion	D44 Vibration	D45 Pinpricking	D46 Clamping the Tail	D47 Vibration	D48 Swimming in Iced Water	D49 Day-Night Inversion
D50 Noise	D51 Fasting	D52 Fasting	D53 Pinpricking	D54 Swimming in Iced Water	D55 Noise	D56 Clamping the Tail

Fasting: Not feeding in 48 hours, free access to water, twice; Water Deprivation: Not watering in 24 hours, free access to food, twice; Day-Night Inversion: covering the cages with lightproof but venting cloth and lighting the cages all the night, twice; Night Lighting: Keeping lighting the whole day, Twice; Swimming in Iced Water: putting ice in cold water, making the temperature 4 degrees Celsius, and then putting the wistar rats in the water for 5 minutes, twice; Clamping the Tail: Clamping the tails of the wistar rats until they struggle and scream, twice; Vibration: Vibrating the cage in order to make the wistar rats can not stand steadily, twice; Pinpricking: using the syringe needle to prink the body of the wistar rats, twice; Noise: Using iron lids to hit the cage until the wistar rats spook or squeeze, twice; Wet Feeding: Wetting the wooden meals and making the wistar rats live in them for 24 hour, once.

### 2.4 Observational Index

2.4.1 Liver pathology test: HE stained method, Microscopic Observation.

2.4.2 Serum TG, Liver TG: GPO-PAP Method.

2.4.3 Serum Ch, HDL-C:Enzyme Colorimetric Method

2.4.4 Serum ALT, AST: Reitman-Frankel methods.

### 2.5 Statistical analysis

Results are expressed as mean ± standard error of the mean (S.E.M.) for n independent observations as indicated. Statistical differences between mean values of groups have been determined using one way analysis of variance (ANOVA) followed by a Dunnett's post-significance test for comparison of multiple means using the SPSS version 11.5. The level of significance was set at P < 0.05.

## 3. Results

### 3.1 Comparison of serum and blood lipid metabolism index between each group

The level of TG, Ch and HDL-C of the wistar rats in group A is separately 0.23 ± 0.05 mmol/L, 1.84 ± 0.45 mmol/L, 1.02 ± 0.19 mmol/L. Compared with the ones of group A, the wistar rats in group B have higher level of TG, 0.79 ± 0.17 mmol/L, higher level of Ch, 15.91 ± 3.59 mmol/L, which show extremely remarkable statistic differences (P < 0.01), and clear lower level of HDL-C, only 0.46 ± 0.07 mmol/L, which also shows extremely remarkable statistic differences (P < 0.01).

The level of TG, Ch and HDL-C of the wistar rats in group C is separately 1.43 ± 0.79 mmol/L, 19.21 ± 1.79 mmol/L and 0.39 ± 0.04 mmol/L Compare with the indexes of group C, the level of Ch and TG are higher and HDL-C is lower. All the differences between the indexes of these two group shows extremely remarkable statistic differences (P < 0.01) (Table 2).

**Table 2:** Comparison of serum and blood lipid metabolism index between each group

Group	TG (mmol/L)	Ch (mmol/L)	HDL-C (mmol/L)
Group A	0.23 ± 0.05	1.84 ± 0.45	1.02 ± 0.19
Group B	0.79 ± 0.17##	15.91 ± 3.59##	0.46 ± 0.07##
Group C	1.43 ± 0.79+,##	19.21 ± 1.79+,##	0.39 ± 0.04++,##

compared with group A, ##P < 0.01; compared with group B, +P < 0.05, ++P < 0.01.

### 3.2 Comparison of serum inflammation index between each group

Compared with the activities of ALT and AST of group A, which are 4.67 ± 1.64 U/L and 18.85 ± 3.90 U/L, the activities of ALT and AST of group B are much more higher, the results of these two indexes are 32.60 ± 9.53 U/L and 42.66 ± 11.78 U/L. We can find remarkable statistic difference (P < 0.01). Compared with the activities of ALT and AST of group B, the activities of ALT and AST of group C are much higher, rising to 53.93 ± 14.73 U/L and 99.61 ± 24.98 U/L, which states great statistic difference (P < 0.01) (Table 3).

**Table 3:** Comparison of serum and blood lipid metabolism index between each group

Group	ALT (U/L)	AST (U/L)
Group A	4.67 ± 1.64	18.85 ± 3.90
Group B	32.60 ± 9.53##	42.66 ± 11.78##
Group C	53.93 ± 14.73++,##	99.61 ± 24.98++,##

compared with group A, ##P < 0.01; compared with group C, ++P < 0.01.

### 3.3 Comparison of liver TG content between each group

Compared with the content of liver TG of group A, 0.41 ± 0.11 mmol/L, the content of liver TG of group B, 0.87 ± 0.25 mmol/L, is much higher as compared with the contnet of liver TG of group A, which states notable statistic difference (P < 0.01). And the last, the content of liver TG of group C is 0.79 ± 0.18 mmol/L. There is no obvious statistic difference between group B and group C. (Table 4).

**Table 4:** Comparison of liver TG content between each group

Group	TG (mmol/L)
Group A	0.41 ± 0.11
Group B	0.87 ± 0.25##
Group C	0.79 ± 0.18##

compared with group A, ##P < 0.01.

### 3.4 Comparison of Liver histopathology between each group

**Figure 1:**
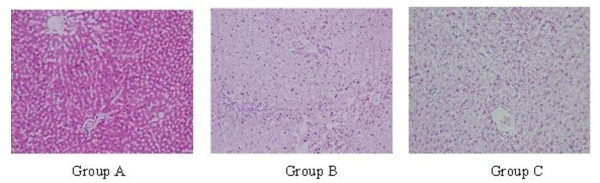
**Rats' HE staining of liver, × 200**.

The hepatic lobules of the wistar rats in group A was complete, the hepatic cord was neat, the hepatic sinusoid was clear and the hepatic cells had irregular polygon or oval somata with ample cytoplasm and big and round karyon in the middle; In the result of the group B, the hepatic cord is neat, the hepatic sinusoid growed bigger and the inflammatory cell infiltration is obvious, Figure 1.

## 4. Discussion

The commonly encountered disease frequently and encountered disease of Lipid Metabolism Disorder are the abnormal high level of the blood TG and CH and decrease of the HDL-C level. Most of the previous Lipid Metabolism Disorder animal model used only one pathogenic factor in the experiment. That is not completely same with the clinical characteristics. Therefore, exploring and establishing a a animal models which simulate the clinical Lipid Metabolism Disorder with multiple nosogenesis is critical for the maintenance of this disease

This research finds that Simple high-fat-diet will stimulate the activities of ALT and AST, CH level, serum TG level and liver TG level to increase, and the HDL-C level to decrease. And these influences will result in Lipid Metabolism Disorder. And the combination of chronic stress and high-fat-diet will make the symptoms of the Lipid Metabolism Disorder on the wistar rats more serious. This shows that these two has the function of stimulating Lipid Metabolism Disorder coordinately, which is more accord with the disease characteristics of human beings and of great research significance.

The research shows that hereditary factor and improper diet is the main factors that cause Lipid Metabolism Disorder [6,7]. As we concerned, in the modern society, the life pace becomes keep increases and the social competition gets more intense day by day. The growing pressure makes people easier to produce emotional change such as sadness, anger, panic and sullenness. And it can also cause the dysfunction of the organs, resulting in Lipid Metabolism Disorder.

So, the model that chronic stress functions coordinating with high fat diet to induce the Lipid Metabolism Disorder of wistar rats is a multiple factor models and is in accordance with the disease characteristics of human beings.

## Competing interests

The authors declare that they have no competing interests.

## Authors' contributions

CSD conceived, designed and coordinated the work, as well as prepared the manuscript. LMT was involved in the co-design of the work as well as the draft of the manuscript. LYH carried out analytical work; ZHH carried out analytical work and contributed in drafting the manuscript. LJ carried out analytical and statistical analysis. All authors read and approved the final manuscript.
